# A Doctors' Union

**Published:** 1896-10-03

**Authors:** 


### The Institutional Workshop.

### A DOCTORS' UNION.

In a long and well thought-out article the *Times* lays down the consideration which should be borne in mind in any attempt by means of a "union" to prevent the further degradation of the profession.

It is in the first place pointed out that the present system of free and unrestricted competition, of struggling for employment by means of advertisement and underselling, is as harmful to the well-being of the patient as it is to the character of the doctor who takes part in it. A distinction has to be drawn, and is drawn, between the work of medical men and other forms of labour which can be readily supervised and the value of which can be accurately determined.

No patient it is said, can estimate the character of the work that his doctor is doing for him—not even by dying can he prove the inappropriateness of the treatment. The patient is as directly interested in his doctor's honesty as he is in his professional skill, and the public ought to see that, since smallness of fee is no proof of cheapness of attendance, it is in every way the loser by the degradation that is only too surely overtaking the profession as the result of the uncontrolled competition amongst its members. This competition shows itself in its worst form in relation to certain commercial associations which out of the proceeds of small weekly payments from the poorer classes provide themselves with a handsome dividend, the patient with medical attendance and a costly funeral, and the undertaker and the doctor with the smallest remuneration that they can be persuaded to accept. But what is medical attendance worth under such circumstances? I is clearly for the good of the public that such down-grade competition should be put an end to.

In devising remedies the *Times* shows what could be done by a widespread union among medical men for the sole object of regulating professional remuneration.

It must not be forgotten that, to the great mass of workers and bread-winners, to be sick is to be poor, and therefore that whatever else is done the provident principle of payment for medical attendance must be accepted. There are now, and there must be in the future, four classes of patients—the private patient, the provident or club patient, the charity patient, and the pauper patient, and by a proper union among the doctors there should be no difficulty in so adjusting the fees from the one class, the subscriptions from the other, and the relations of the charities to both, that the competition within the profession should take the form of giving the best service instead of snatching even at the smallest fee.

The *Times* truly says, "the doctors have but to join hands for their mutual advantage, and the union could be formed to-morrow," and there can be do doubt that such a professional union would be for the general good. But will they do it?

